# Mentalizing Abilities in Major Depressive Disorder and Borderline Personality Disorder: Measuring Hypermentalization and Implicit Mentalization with the Hungarian Version of the MASC

**DOI:** 10.1192/j.eurpsy.2024.1095

**Published:** 2024-08-27

**Authors:** D. Karakas-Török, E. Fábi, M. Szennai, C. Csuta, O. Kelemen, T. Tényi, B. Czéh, M. Simon

**Affiliations:** ^1^Department of Psychiatry and Psychotherapy, Medical School, University of Pécs, Pécs; ^2^Albert-Szentgyörgyi Medical School, Department of Behavioral Sciences, University of Szeged, Szeged; ^3^Department of Psychiatry, Bács-Kiskun County Hospital, Kecskemét; ^4^Department of Laboratory Medicine, Medical School; ^5^Neurobiology of Stress Research Group, Szentágothai János Research Centre, University of Pécs, Pécs, Hungary

## Abstract

**Introduction:**

Borderline Personality Disorder (BPD) is the most common personality disorder in psychiatric care. BPD often co-occurs with Major Depressive Disorder (MDD). Both BPD and MDD are associated with various impairments of social functioning. Among these, mentalizing disturbances are the most extensively studied.

**Objectives:**

The Movie for the Assessment of Social Cognition (MASC) is an ecologically valid video-based test, which is suitable for measuring both hypermentalization and implicit mentalization. Based on the literature, it is sensitive enough to detect mild deficits in mentalization capacities. In this study, we investigated mentalization deficits with a special focus on implicit mentalization and hypermentalization in patients with MDD and MDD+BPD with a set of well-established mentalization tests including MASC.

**Methods:**

We examined patients with MDD (n=43) during the depressive episode. A subgroup of these patients was also diagnosed with BPD (MDD+BPD group; n=23), the other group had no comorbid personality disorder (MDD group; n=20). We assessed the patients’ mentalization abilities using the Hungarian version of the Reading the Mind in the Eyes test, the Faux Pas test, and the MASC test. Additionally, symptom scales (measuring the severity of anxiety, and depression), WAIS (Wechsler Adult Intelligence Scale), the Childhood Trauma Scale, as well as scales measuring affect regulation and attachment were used during the assessment.

**Results:**

There were no differences between the two groups in terms of age, IQ, or the severity of depression and anxiety. The MDD+BPD group exhibited significantly poorer performance in the MASC total mentalization score (MW U=118, df=1,41, p< 0,001), as well as in the hypermentalization score (MW U=98,5, df=1,41, p< 0,001). The MDD+BPD group achieved significantly lower results on the emotion recognition and mentalization measures in the RMET test (t=2,883, df=1,41, p< 0,001). The MDD+BPD group performed significantly worse on the Faux Pas test measuring mentalization (MW U=144,5, df=1,41, p< 0,001). In the whole sample, MASC performance correlated with overall IQ.

**Conclusions:**

The MASC, RMET, and Faux Pas tests show a consistent trend and indicate significant differences between the mentalization abilities of MDD+BPD and MDD patients. Our findings are in line with data in the literature: BPD patients’ implicit mentalization with a predominance of hypermentalization is impaired. This impairment is detectable when we compare their performance with MDD patients without BPD. In the future, a larger sample size, additional tests, and the inclusion of a control group are needed to further investigate MDD and MDD+BPD patients’ mentalizing deficits. However, our results emphasize the significance of mentalization-based therapies in the therapy of patients with BPD and depression.

**Disclosure of Interest:**

None Declared

